# Outcomes of Hypofractionated Stereotactic Radiotherapy for Small and Moderate-Sized Brain Metastases: A Single-Institution Analysis

**DOI:** 10.3389/fonc.2022.869572

**Published:** 2022-04-04

**Authors:** Michael Yan, Osbert Zalay, Thomas Kennedy, Timothy E. Owen, James Purzner, Shervin Taslimi, Teresa Purzner, Ryan Alkins, Nikitha Moideen, Andrea S. Fung, Fabio Y. Moraes

**Affiliations:** ^1^ Department of Oncology, Division of Radiation Oncology, Kingston Health Sciences Centre, Queen’s University, Kingston, ON, Canada; ^2^ Division of Neurosurgery, Department of Surgery, Kingston Health Sciences Centre, Queen’s University, Kingston, ON, Canada; ^3^ Department of Oncology, Division of Medical Oncology and Hematology, Kingston Health Sciences Centre, Queen’s University, Kingston, ON, Canada

**Keywords:** brain metastases, radiosugery, radiation oncology, radionecrosis, neurosurgery

## Abstract

**Background:**

Stereotactic radiosurgery (SRS) is the standard treatment for limited intracranial metastases. With the advent of frameless treatment delivery, fractionated stereotactic radiotherapy (FSRT) has become more commonly implemented given superior control and toxicity rates for larger lesions. We reviewed our institutional experience of FSRT to brain metastases without size restriction.

**Methods:**

We performed a retrospective review of our institutional database of patients treated with FSRT for brain metastases. Clinical and dosimetric details were abstracted. All patients were treated in 3 or 5 fractions using LINAC-based FSRT, did not receive prior cranial radiotherapy, and had at least 6 months of MRI follow-up. Overall survival was estimated using the Kaplan–Meier method. Local failure and radionecrosis cumulative incidence rates were estimated using a competing risks model with death as the competing risk. Univariable and multivariable analyses using Fine and Gray’s proportional subdistribution hazards regression model were performed to determine covariates predictive of local failure and radionecrosis.

**Results:**

We identified 60 patients and 133 brain metastases treated at our institution from 2016 to 2020. The most common histologies were lung (53%) and melanoma (25%). Most lesions were >1 cm in diameter (84.2%) and did not have previous surgical resection (88%). The median duration of imaging follow-up was 9.8 months. The median survival for the whole cohort was 20.5 months. The local failure at 12 months was 17.8% for all lesions, 22.1% for lesions >1 cm, and 13.7% for lesions ≤1 cm (p = 0.36). The risk of radionecrosis at 12 months was 7.1% for all lesions, 13.2% for lesions >1 cm, and 3.2% for lesions ≤1 cm (p = 0.15).

**Conclusions:**

FSRT is safe and effective in the treatment of brain metastases of any size with excellent local control and toxicity outcomes. Prospective evaluation against single-fraction SRS is warranted for all lesion sizes.

## Introduction

Brain metastases are a frequent manifestation of metastatic cancers, with an estimated incidence rate of 10–20 per 100,000 persons per year in the general population (1, 2). Given the low penetrance of traditional chemotherapies across the blood–brain barrier, radiotherapy is often the most important treatment option (3, 4). Although recent advances in systemic therapies show promise of improved penetrance with tyrosine kinase inhibitors (TKIs) and immunotherapies, only a subset of patients benefit from these new treatment options, with objective response rates ranging from 50% to 80% (5–8). Conversely, radiotherapy is not limited by anatomical barriers, and stereotactic radiosurgery (SRS) has been shown to effectively control metastases with 1-year local control rates ranging from 60% to 90%, depending on lesion size (9).

SRS has been established as standard therapy in the setting of limited brain metastases. Several randomized trials have demonstrated equivalent local control outcomes compared to whole-brain radiotherapy (WBRT) treatment while sparing neurocognitive toxicity (10). Historically, single-fraction treatment was given with de-escalation of the prescription dose based on increasing lesion size (11). Fractionated stereotactic radiotherapy (FSRT) has been shown to result in superior local control and radiation necrosis rates for lesions larger than 2 cm in diameter (12, 13).

Given the demonstrated advantages in local control and toxicity for large metastases, we hypothesize that smaller lesions may also show improved outcomes when treated with FSRT. At our institution, we routinely treat all metastases with FSRT without a minimum size limit. The objective of this study is to report our single-institution experience, with a focus on comparing outcomes based on lesion size.

## Methods

### Data Sources and Treatment Details

We performed a local ethics board-approved retrospective review of patients with *de novo* brain metastases treated between May 2016 and March 2020 at the Cancer Centre of Southeastern Ontario. All patients were treated using LINAC-based FSRT and received 18–32.5 Gy in 3 or 5 consecutive daily fractions. We attained clinical and treatment characteristics of individual patients and lesions. Radiosensitive histologies consisted of breast, head and neck, gynecological, and lung cancer primaries. Radioresistant tumors included melanoma, gastrointestinal site, and non-prostate genitourinary primaries. Dose prescriptions were not adapted based on histology.

As per institutional policy, all patients were of Eastern Cooperative Oncology Group (ECOG) performance status ≤2 and had ≤10 intracranial metastases. Target lesions with at least 6 months of MR follow-up and previous radiotherapy involving the target site were excluded from the current analysis. Institutional review board approval was attained for the conduct of this study.

Patients were CT-simulated using 1-mm image slices with immobilization using a thermoplastic mask in the supine position. Volumetric MRIs with a slice thickness of 1–2 mm were fused with the planning image set using rigid registration, utilizing the T1-weighted post-gadolinium and T2-weighted sequences. For intact lesions (previously untreated; with neither surgery nor radiation), gross tumor volume (GTV) was delineated using the T1 post-gadolinium sequence. For postoperative cavities, the enhancing cavity was delineated using the T1 post-gadolinium sequence as the GTV, and a subsequent expansion measuring 2–3 mm is added to incorporate any microscopic disease as a clinical target volume (CTV). Finally, a 2-mm isotropic expansion was applied to the GTV or CTV for intact lesions and cavities to generate a planning target volume (PTV). Radiotherapy was delivered using Varian TrueBeam linear accelerators (LINAC) equipped with micro multi-leaf collimators (MLC) using a flattening filter-free beam of 6-MV energy and volumetric modulated arc therapy (VMAT) technique. All patients were treated on hexapod couches, with a 1-mm tolerance for positional error. Daily cone-beam CT (CBCT) was used for treatment setup verification.

All patients were treated in 3 or 5 daily fractionation schedules, with the dose selection influenced by tumoral volume and location. Common doses include 3 fractions at 21, 24, and 27 Gy and for 5-fraction regimens at 25, 27.5, 30, and 32.5 Gy. Larger tumors generally received a 5-fraction treatment, although the prescription dose was left at the discretion of the treating oncologist. All plans were normalized so that the prescription dose covered at least 95% of the PTV. A conformity index of ≤1.2, defined by the ratio of the prescription isodose volume to the PTV volume, was maintained. The gradient index pertaining to the containment of the dose falloff between the 100% and 50% isodose line was required to be <2 cm. Doses were converted to biologically effective dose (BED) through the following formula:


BED=nd(1+dαβ)

where *n* represents the number of fractions, *d* is the dose per fraction, and *α*/*β* represents the cellular sensitivity to fractionation corresponding to the linear-quadratic model, which is defined as 10 for tumoral cells.

Follow-up was at the discretion of the treating oncologist but usually consisted of brain MRI every 3 to 4 months. Large lesions were defined as those >1 cm in diameter; otherwise, they were categorized as small.

### Outcome Definition and Statistical Analysis

The primary outcomes are local failure and radiation necrosis (RN). Events of local failure were defined by pathological confirmation of significant proportions of viable tumor in the event of surgery or based on imaging using the Response Assessment in Neuro-Oncology Brain Metastases (RANO-BM) criteria (14). Progression of target lesions is defined as a 20% increase in the sum of the longest diameter with at least a 5-mm total increase or, in the case of lesions <1 cm, a 3-mm total increase. Local failure was also defined by the interval development of perilesional leptomeningeal disease. RN was defined as radiographic progression of disease followed by stabilization or regression in the absence of oncologic directed therapies, or as suggested by a low relative cerebral blood volume on MR perfusion-weighted imaging. RN could be symptomatic or asymptomatic, with time to RN defined at the time of the first scan showing target lesion growth.

Baseline variables were summarized using descriptive statistics. The Mann–Whitney U test and chi-square tests were used to determine significant differences in characteristics of large and small lesions for continuous and categorical variables, respectively.

All dates were calculated from the end of FSRT treatment. The Kaplan–Meier method was used to estimate the survival function for all patients up to the date of death from any cause or last known follow-up. Patients who were lost to follow-up or received whole-brain radiotherapy after FSRT were censored. Cumulative incidence functions (CIFs) were estimated using the Aalen–Johansen method for both local failure and RN using the subdistribution hazards method, with death from any cause as a competing risk. Differences between CIFs were compared using Gray’s test. Fine and Gray’s method for estimating proportional subdistribution hazards in a competing risks framework was used to compute hazard ratios (HRs) and determine significant predictors for the primary endpoints in multivariable models. Again, death from any cause was the competing risk. Clinically significant variables were chosen *a priori for* each endpoint. All tests were two-sided, with a p-value <0.05 deemed to be significant. All analyses were conducted using Python version 3.6.5 (Python Software Foundation) and R 3.6.

## Results

### Patient and Lesion Characteristics

A total of 133 brain metastases in 60 patients were treated with FSRT, with a median tumor size of 1.3 cm (range 0.57–4.80), with 15.8% of lesions being ≤1 cm in diameter. Patient and lesion characteristics are summarized in **Table 1**. The mean age for the whole cohort was 65 (range 33–88), and most patients were female (61.7%). As expected, the most common histologies were lung cancer (53.4%) and melanoma (24.9%). Most treated lesions were radiosensitive (70.1%) and larger than 1 cm in size (84.2%). More than half of patients did not have prior systemic therapy (52.8%) and did not have prior surgical resection (88%). Compared to lesions ≤1 cm in diameter, larger lesions were more likely to have been in patients who received systemic therapy post-FSRT course (p = 0.002). Similarly, all post-operative FSRT was performed in lesions initially larger than 1 cm (p = 0.04).

**Table 1:** Baseline patient and lesion characteristics.

Characteristic	All patients	Lesion diameter ≤1 cm (n_L_ = 21)	Lesion diameter >1 cm (n_L_ = 112)	p-Value
Patient (n = 60)				
Sex (n, %)				
Male	23 (38.3)	N/A	N/A	N/A
Female	37 (61.7)			
Age, years (mean, range)	65 (33–88)	N/A	N/A	N/A
Number of metastases (%)				
1–3	53 (88.3)	N/A	N/A	N/A
4+	7 (11.7)			
Primary site (n, %)				0.71
Lung	71 (53.4)	9 (42.9)	62 (55.4)	
Melanoma	33 (24.9)	7 (33.3)	26 (23.2)	
Gastrointestinal	1 (0.8)	0 (0)	1 (0.9)	
Gynecological	4 (3.0)	0 (0)	4 (3.6)	
RCC	3 (2.3)	0 (0)	3 (2.7)	
Breast	18 (13.5)	5 (23.8)	13 (11.6)	
Other	3 (2.4)	0 (0)	3 (2.7)	
Lesion (n = 133)				
Sensitivity to radiation				0.66
Radiosensitive	94 (70.1)	14 (66.6)	80 (71.4)	
Radioresistant	39 (29.9)	7 (33.3)	32 (28.6)	
Prior systemic therapy (n, %)				1.00
Chemotherapy	17 (12.8)	0 (0)	17 (15.2)	
TKI/immunotherapy	41 (30.8)	9 (42.9)	32 (28.6)	
Both	12 (9.0)	2 (9.5)	10 (8.9)	
None	69 (52.8)	10 (47.6)	59 (52.7)	
Post-RT systemic therapy (n, %)				0.002
Chemotherapy	35 (26.3)	2 (9.5)	33 (29.5)	
TKI/immunotherapy	48 (36.1)	3 (14.3)	45 (40.2)	
Both	26 (19.5)	1 (4.8)	25 (22.3)	
None	50 (37.6)	15 (71.4)	35 (31.3)	
Surgical resection pre-FSRT (n, %)				0.04
Yes	16 (12)	0 (0)	16 (14.3)	
No	117 (88)	21 (100)	96 (85.7)	

IQR, interquartile range; TKI, tyrosine kinase inhibitor; FSRT, fractionated stereotactic radiotherapy; RCC, renal cell carcinoma; N/A, not applicable.

Dosimetric outcomes are shown in **Table 2**. The median PTV diameter amongst all lesions was 2 cm (interquartile range [IQR], 1.8), and the median volume was 2 cc (IQR, 11.2). The median BED_10_ of the D95 to the PTV was 32.5 Gy (IQR 0.5), and the median V12 was 55 cc (IQR, 94.7). There was no significant difference in BED_10_ or V12 between large and small lesions.

**Table 2:** Dosimetric characteristics.

Characteristic	All lesions (n = 133)	Lesion diameter ≤1 cm (n = 21)	Lesion diameter>1 cm (n = 112)	p-Value
PTV diameter, cm (median, IQR)	2 (1.8)	0.9 (0.2)	2 (2)	<0.001
PTV volume, cm^3^ (median, IQR)	2 (11.2)	0.4 (0.2)	3 (13)	0.05
D95 dose, BED_10_, (median, IQR)	33 (0.1)	32.6 (0.1)	32 (1)	0.13
V12Gy, cm^3^ (median, IQR)	55 (94.7)	22.1 (84.9)	64 (108)	0.08

PTV, planning target volume; IQR, interquartile range; BED, biologically effective dose.

**Figure 1:**
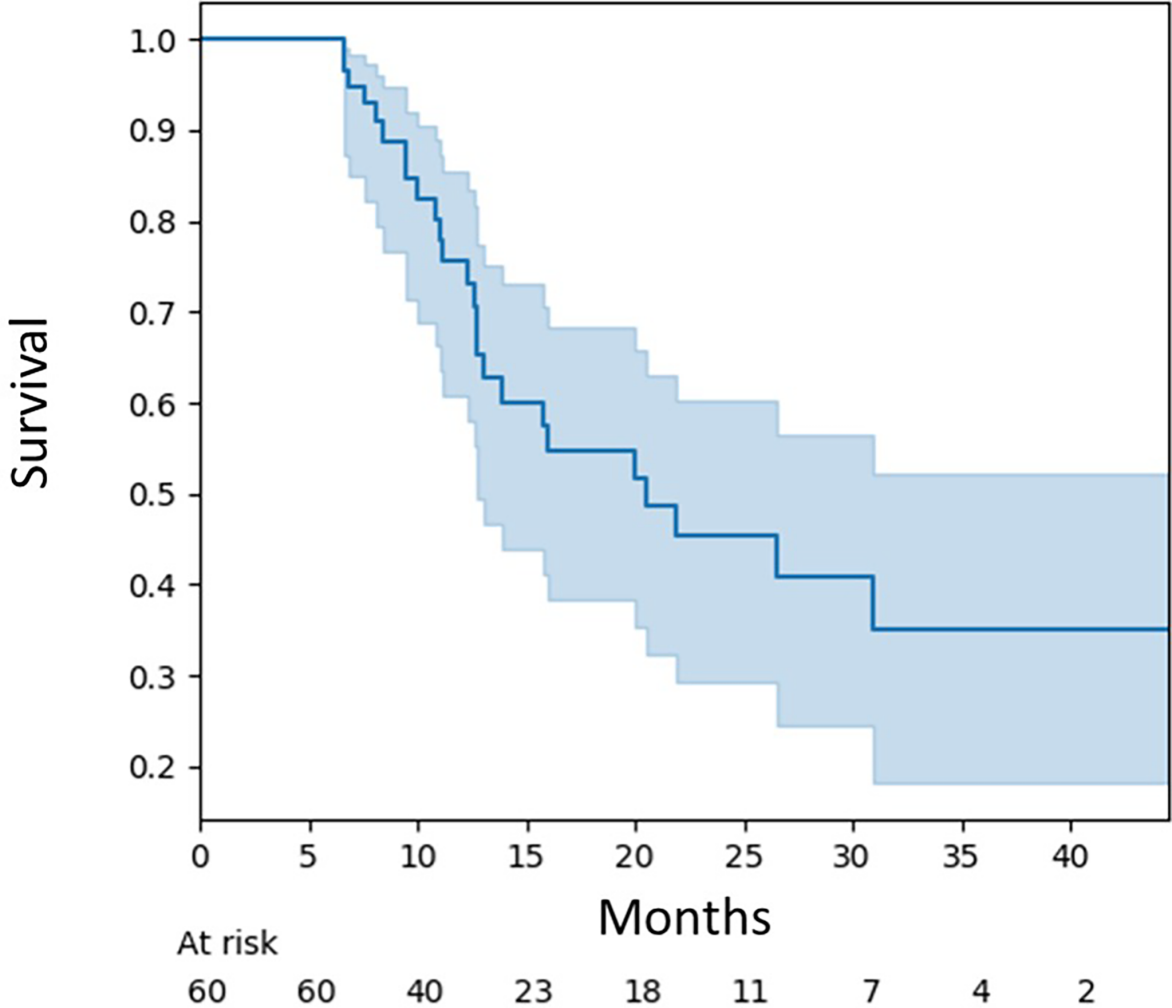
Overall survival for all patients.

The median brain MRI follow-up for the cohort was 9.8 months (range 6.0–43.5). The median overall survival for this patient cohort was 20.5 months (95% CI, 12.6–65.9) (**Figure 1**).

### Local Failure and Radiation Necrosis

**Figure 2:**
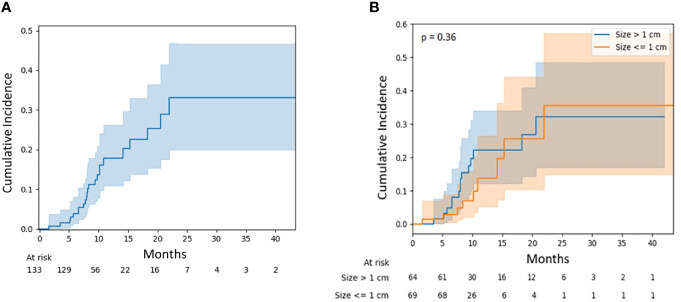
**(A)** Local failure for all lesions. **(B)** Local failure for lesions stratified by diameter: ≤1 vs. >1 cm.

The local failure rate was 17.8% (95% CI, 10.8–26.2) at 12 months and 32.4% (95% CI, 19.8–46.1) at 24 months (**Figure 2A**). The risk of local failure was not significantly different between large (>1 cm) and small lesions (≤1 cm) (p = 0.36) and was 22.1% (95% CI, 12.1–34.0) and 13.7% (95% CI, 5.1–26.5) at 12 months for these two cohorts, respectively (**Figure 2B**). No statistically significant associations were observed on univariable or multivariable regression between surgery, D95 BED_10_ dose, and lesion size with risk of failure.

**Figure 3:**
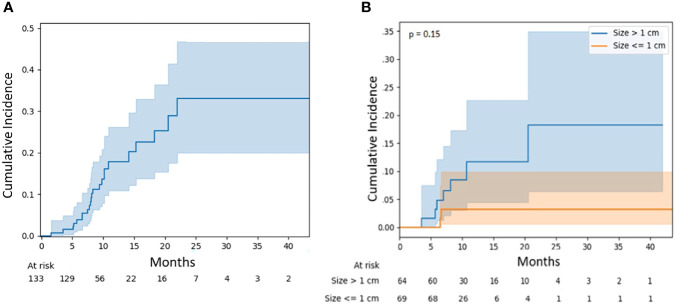
**(A)** Radionecrosis for all lesions. **(B)** Radionecrosis for lesions stratified by diameter: ≤1 vs. >1 cm.

Similarly, the radionecrosis rate was 7.1% (95% CI, 3.1–13.3) at 12 months and 13.2% (95% CI, 5.5–24.4) at 24 months (**Figure 3A**). There was no significant difference in necrosis rates between large and small lesions (p = 0.15), although the 12-month risk was numerically higher for larger lesions at 11.7% (95% CI, 4.5–22.6) versus 3.2% (95% CI, 0.6–9.8) (**Figure 3B**). Again, there was no statistically significant association observed between independent variables and the risk of radionecrosis (**Table 3**).

**Table 3:** Univariable and multivariable analyses for local failure and radiation necrosis.

Factor	Local failure				Radiation necrosis			
	UVA—HR (95% CI)	p-Value	MVA—HR (95% CI)	p-Value	UVA—HR (95% CI)	p-Value	MVA—HR (95% CI)	p-Value
D95 PTV dose ≥Median BED <Median BED (ref)	0.63 (0.27–1.47)	0.29	0.65 (0.28–1.50)	0.31	NI	NI	NI	NI
Dmax PTV dose ≥Median BED <Median BED (ref)	NI	NI	NI	NI	4.25 (0.83–21.7)	0.08	3.91 (0.51–30.1)	0.19
Tumor size >1 cm ≤1 cm (ref)	1.47 (0.64–3.37)	0.36	1.74 (0.71–4.28)	0.23	3.04 (0.67–13.7)	0.15	2.12 (0.27–16.9)	0.48
V12 brain ≥Median <Median (ref)	NI	NI	NI	NI	2.08 (0.53–8.18)	0.29	0.81 (0.11–5.81)	0.83
Previous surgery Yes No (ref)	0.53 (0.12–2.32)	0.40	0.43 (0.08–2.2)	0.31	2.76 (0.7–10.9)	0.15	2.07 (0.39–10.9)	0.39

BED, biologically effective dose; UVA, univariable analysis; MVA, multivariable analysis; HR, hazard ratio; NI, not included.

## Discussion

Our study reports favorable outcomes following FSRT for intact brain metastases using 3- and 5-fraction schedules. We report a 12-month local control rate of 82.2% for all lesions, and in the subset of patients with lesions <1 cm (small lesions), an even better control rate of 86.8% was observed. The 12-month incidence of RN was low at 7.1% in the overall cohort, and when limited to small lesions, it was only 3.2%. The strengths of our study include a minimum of 6 months of MRI follow-up for each lesion, detailed clinical and dosimetric detail collection, and homogenous application of institutional radiosurgery protocol. Our results support FSRT as a safe and effective treatment option in patients with limited brain metastases.

Radiosurgery was first developed by Lars Leksell over 60 years ago, performed using the Gamma Knife machine (15). However, with the advent of flattening filter-free technology, micro-MLC leaves, and hexapod treatment couches, gantry-based LINAC SRS has enabled the widespread adoption and availability of treatment for focal brain metastases. Furthermore, LINAC-based SRS circumvents the need for a stereotactic frame with thermoplastic mask-based immobilization, allowing for hypofractionated treatment. Hypofractionation takes advantage of radiobiological principles to improve the therapeutic ratio and has been associated with better control and a lower risk of RN compared to single-fraction treatment for larger metastases (12, 13, 16). A larger dose per fraction results in greater BED to normal tissues for the same tumoral BED, given their lower *α*/*β* value by comparison. Fractionation therefore narrows the gap in the therapeutic ratio by reducing the dose per fraction (17). The literature is scarce, however, regarding the use of FSRT for smaller intracranial metastases.

The outcomes of our study are consistent with those reported in the literature. Minniti et al. presented one of the first series of 138 patients with brain metastases >2 cm treated with FSRT. They reported a 12-month local control rate and RN of 81% and 9%, respectively. When compared to a cohort of patients who received single-fraction SRS, they found a significantly lower risk of local failure and RN in patients treated with FSRT, before and after propensity score adjustments (13). Lehrer et al. performed a meta-analysis of 24 studies that reported on metastases >2 cm, including a total of 1,887 brain metastases. For lesions 2–3 cm, the local control rate at 12 months was 92.9% with FSRT, and the RN rate was 7.3% (12).

There is no consensus on the definition of small brain metastases, although traditionally, FSRT has been reserved for lesions >2 cm (12, 18). A unique aspect of our analysis is that a considerable proportion of the treated metastases (nearly 16%) were smaller than 1 cm. Compared to larger lesions, those <1 cm had a lower numerical rate of failure as well as RN, although the differences were not significantly different. However, this is likely due to small sample sizes and low study power. Within the FSRT literature, outcomes of small metastases are heterogeneous but excellent overall. Marcrom et al. compared 182 intact metastases and found that lesions <3 cm in diameter had a 95% control rate at 12 months compared to 75% for larger lesions with FSRT. They also observed increased toxicity with each 1 cm increase in lesion diameter (HR 2.45, p = 0.04) (19). Similarly, Mengue et al. observed improved local control for lesions <2.5 cm compared to larger (p = 0.02). Radionecrosis occurred in only 5% of patients, with a median size of 2.3 cm with FSRT (20).

Few direct comparative studies between single-fraction SRS and FSRT are currently published for small brain metastases. Samanci et al. compared single-fraction SRS and FSRT for the treatment of 208 small brain metastases <4 cc in size, treated with the Gamma Knife Icon. They observed excellent 6-month local control rates of 99% for both single-fraction and FSRT. However, radionecrosis was only observed in the single-fraction SRS cohort, with an incidence of 2%, and not in the patients who received FSRT (21). A prospective series of 334 intact metastases, of which 60% were ≤2 cm in diameter, were treated with a 5-fraction schedule. The reported 2-month local control rate was 76.2%, and the adverse radiation effect (ARE) rate was 15.6%. A prescription dose of >30 Gy was associated with improved local control compared to lower doses (HR 1.62, p = 0.03) (16). In a volumetric, comparative study of FSRT and SRS, Putz et al. determined similar 12-month rates of local control at 68.6% and 65.4% for FSRT and SRS, respectively, while radionecrosis was 0% versus 9.6% for the same groups (22). Although our study was not comparative between single-fraction SRS and FSRT, our local control and toxicity outcomes compare similarly to these literature outcomes.

The management of brain metastases is multimodal. Systemic therapy options are rapidly evolving and have been associated with improvements in survival in patients with metastatic disease on a population level (23, 24). Our understanding of the interplay between radiotherapy and systemic therapies, in particular immunotherapy and targeted agents, is developing. Several trials have suggested brain penetrance of newer generation targeted agents and immunotherapy, with observed objective response rates as high as 80% (25–27). Safety of radiotherapy and systemic agents is another concern, with evidence suggesting an increased risk of RN with concomitant radiosurgery and immunotherapy (28, 29). Optimal sequencing of radiotherapy and newer generation systemic therapies is an area of active research.

Our study is limited by the usual caveats of retrospective studies. Given the observational nature of the data, confounding variables cannot be controlled for in the observation of the reported HRs. Furthermore, we did not determine any statistically significant associations on regression analysis due to our small sample sizes, thereby limiting study power. Nevertheless, our experience based on robust data supports the safety and efficacy of FSRT for metastases of all sizes.

## Conclusions

LINAC-based FSRT is a safe and effective treatment for limited intracranial metastases. We observed acceptable rates of local control and radionecrosis, particularly in metastases smaller than 1 cm in diameter. With the widespread availability of gantry-based linear accelerators, LINAC-based FSRT is a feasible treatment option that can be readily adopted and implemented by many radiotherapy centers, especially when SRS is not available.

## Data Availability Statement

The raw data supporting the conclusions of this article will be made available by the authors, without undue reservation.

## Ethics Statement

The studies involving human participants were reviewed and approved by Queens University/Kingston Health Sciences Centre. The ethics committee waived the requirement of written informed consent for participation.

## Author Contributions

Study conception: MY, NM, and FM. Study design: MY, NM, and FM. Data acquisition: MY, OZ, TK, and NM. Data quality and statistical analysis: MY, OZ, TK, and NM. Manuscript preparation: MY, OZ, TK, TO, JP, TP, RA, AF, and FM. Manuscript editing and review: all authors.

## Conflict of Interest

The authors declare that the research was conducted in the absence of any commercial or financial relationships that could be construed as a potential conflict of interest.

## Publisher’s Note

All claims expressed in this article are solely those of the authors and do not necessarily represent those of their affiliated organizations, or those of the publisher, the editors and the reviewers. Any product that may be evaluated in this article, or claim that may be made by its manufacturer, is not guaranteed or endorsed by the publisher.
